# Reduced glutathione as a physiological co-activator in the activation of peptidylarginine deiminase

**DOI:** 10.1186/s13075-016-1000-7

**Published:** 2016-05-05

**Authors:** Dres Damgaard, Mads Emil Bjørn, Maria A. Steffensen, Ger J. M. Pruijn, Claus H. Nielsen

**Affiliations:** Institute for Inflammation Research, Center for Rheumatology and Spine Diseases, Copenhagen University Hospital, Rigshospitalet, Copenhagen, Denmark; Section for Periodontology, Microbiology and Community Dentistry, Department of Odontology, Faculty of Health and Medical Sciences, University of Copenhagen, Copenhagen, Denmark; Department of Haematology, Roskilde Hospital, Roskilde, Denmark; Department of Biomolecular Chemistry, Institute for Molecules and Materials, Radboud Institute for Molecular Life Sciences, Radboud University Nijmegen, Nijmegen, The Netherlands

**Keywords:** Peptidylarginine deiminase, Reduced glutathione, Rheumatoid arthritis, Citrullination, Synovial fluid

## Abstract

**Background:**

Citrullination catalysed by peptidylarginine deiminases (PADs) plays an important pathogenic role in anti-citrullinated protein antibody (ACPA)-positive rheumatoid arthritis (RA) and, possibly, several other inflammatory diseases. Non-physiological reducing agents such as dithiothreitol (DTT) are normally added to the reaction buffer when determining PAD activity in vitro. We investigated the ability of reduced glutathione (GSH), the most abundant intracellular small-molecule thiol in vivo, to activate PADs.

**Methods:**

Activity of recombinant human (rh) PAD2 and PAD4, PADs contained in synovial fluid (SF) samples from RA patients and PADs released from phorbol 12-myristate 13-acetate (PMA)-stimulated cells was measured using an in-house PAD activity assay detecting citrullination of fibrinogen.

**Results:**

No activity of rhPAD2, rhPAD4 or PADs within SF was observed without addition of an exogenous reducing agent. Activity of both recombinant and SF PAD was observed in the presence of 1 mM DTT or 10–15 mM GSH. Following stimulation with PMA, human isolated leucocytes, but not mononuclear cells, released enzymatically active PAD, the activity of which was abolished upon pre-incubation of the cells with the glutathione reductase inhibitor 2-AAPA. No PAD activity was observed in the corresponding supernatants, but addition of exogenous GSH restored activity.

**Conclusions:**

Catalytic activity of PAD requires reducing conditions. GSH meets this requirement at concentrations comparable with those found within cells. Active PAD, reduced by GSH, is released from PMA-stimulated granulocytes, but becomes inactivated in the extracellular space.

## Background

Citrullination refers to the post-translational conversion of protein arginine residues into citrulline residues, a process catalysed by peptidylarginine deiminases (PADs) 1–4 and PAD6 [1]. Citrullination plays an important pathogenic role in anti-citrullinated protein antibody (ACPA)-positive rheumatoid arthritis (RA) [2, 3] and, possibly, in a number of other autoimmune diseases, inflammatory diseases or neurodegenerative conditions, including multiple sclerosis [4], Alzheimer’s disease [5], psoriasis [6], Sjögren’s syndrome [7], type 1 diabetes [8] and chronic obstructive pulmonary disease [9].

Most studies on PAD activity and functional studies of citrullinated proteins have been based on in vitro citrullination using the reducing agent dithiothreitol (DTT) and exogenously added calcium. The calcium dependency of PADs is well described: upon binding of calcium, Cys645 in PAD4 (Cys647 in PAD2) translocates into a position in the catalytic site, where nucleophilic attack on guanidinium groups of target arginines takes place [10, 11]. The PAD requirement for calcium is met in the extracellular space [12] and, under certain circumstances, also intracellularly [13]. The dependency of PADs on reducing agents is less well understood. Reduction of the active site thiol Cys645/647 is likely to precede attack on the guanidinum carbon of arginine. Thus, the redox balance might be an additional regulator for PAD’s catalytic activity. While DTT is a non-physiological synthetic molecule, reduced glutathione (GSH) is a physiological reducing agent that may facilitate PAD activity in vivo. GSH is a linear tripeptide of l-glutamine, l-cysteine and glycine, and contains a sulfhydryl (SH) group on the cysteinyl portion, accounting for its strong electron-donating character. GSH is the most abundant intracellular small-molecule thiol, and is essential for maintaining the thiol status of various molecules. GSH has many biological roles, including protection against reactive oxygen and nitrogen species (ROS/NOS), which are reduced by two GSH molecules forming oxidized glutathione (GSSG) in the process [14]. GSH has been demonstrated in cytosol and in organelles of virtually all cells of the body at the low millimolar range [15], whereas extracellular levels are two to three orders of magnitude lower [16, 17].

Owing to involvement in many cellular functions, it is not surprising that dysregulation of GSH has been associated with various diseases [14]. The glutathione reductase, which converts GSSG to GSH, has been found upregulated in synovial fluid (SF) from RA patients [18], and blood from RA patients contains higher levels of GSH, as well as higher GSH:GSSG ratios, than blood from healthy controls [19].

We hypothesize that, in addition to calcium, reducing agents are required for PADs to become enzymatically active, and that GSH is the major physiological reducing agent in this respect.

Using an assay for citrullination of matrix-bound fibrinogen, we here analyse the impact of GSH on recombinant human PADs (rhPADs), on PADs contained in SF and on PADs released from stimulated human leucocytes.

## Methods

### Cells, serum and SF

Blood samples were obtained from healthy donors attending the Blood Bank at Copenhagen University Hospital, Rigshospitalet, Denmark. All donors were anonymous to the investigators.

One hour after collection, serum was isolated from venous blood drawn into 10 ml dry Vacutainer tubes (BD Bioscience, Brøndby, Denmark) by centrifugation at 400 × *g* for 10 min at 20 °C. Pooled serum from blood group AB-positive donors, henceforward referred to as “AB serum”, was purchased from Sigma-Aldrich (St. Louis, MO, USA).

Cells were isolated from venous blood drawn into 10 ml lithium-heparin tubes (BD Bioscience). Blood leucocytes were isolated after lysis of erythrocytes in heparinized blood by incubation with ammonium chloride (In Vitro As, Fredensborg, Denmark) for 7 min. Mononuclear cells (MNCs) were isolated by gradient centrifugation of heparinized blood using LymphoPrep™ (Axis-Shield, Oslo, Norway). Before use, both cell preparations were washed twice in RPMI 1640, 25 mM Hepes containing 0.42 mM calcium nitrate, l-glutamine and gentamicin (In Vitro As).

SF samples from nine ACPA-positive RA patients, fulfilling the American College of Rheumatology 1987 diagnostic criteria [20], were obtained from Dr Ladislav Senolt, Charles University in Prague, Czech Republic. The study was approved by the Ethics Committee of the Institute of Rheumatology and written informed consents were obtained from all patients prior to initiation of the study. Samples were centrifuged at 1900 × *g* for 10 min to remove cells and were stored at –80 °C prior to analysis.

### Reagents

rhPAD2 and rhPAD4 were produced, purified and defined by means of mass concentration, as described previously [21]. GSH was purchased from Sigma-Aldrich. The glutathione reductase inhibitor (GRI) 2-acetylamino-3-[4-(2-acetylamino-2-carboxyethylsulfanylthiocarbonylamino)phenylthiocarbamoylsulfanyl]propionic acid hydrate (2-AAPA) was purchased from Sigma-Aldrich. Monoclonal mouse anti-citrullinated fibrinogen (clone 20B2; catalogue number MQ13.102) was purchased from ModiQuest (Oss, Netherlands).

### Cell-free assay for PAD activity

Maxisorp plates (Nunc, Roskilde, Denmark) were coated overnight at 4 °C with 100 μl/well of 1.0 μg/ml fibrinogen (Calbiochem, Darmstadt, Germany) in coating buffer (30 mM Na_2_CO_3_, 70 mM NaHCO_3_, pH 9.6). Wells were washed three times and blocked in Tris-buffered saline (TBS) buffer containing 0.05 % Tween-20, pH 7.4, for 20 min at room temperature (RT). Next, the wells were incubated (100 μl/well for 180 min at RT) with: rhPAD2 and/or rhPAD4 (300 ng/ml in 100 mM Tris–HCl, pH 7.5); SF (undiluted 50 μl; diluted 1:2 in 100 mM Tris–HCl, pH 7.5); serum (diluted 1:2 in 100 mM Tris–HCl, pH 7.5); or cell culture supernatants (diluted 1:1 in 100 mM Tris–HCl, 10 mM CaCl_2_, pH 7.5). The reactions took place in the presence of various combinations of rhPAD2/4, DTT (1 mM), EDTA (25 mM) or GSH and CaCl_2_ at various concentrations, as specified in the figure legends. After three washes in washing buffer (PBS, 0.05 % Tween-20, pH 7.4), murine anti-citrullinated fibrinogen antibody (0.5 μg/ml) was incubated for 90 min at RT. After three further washes, wells were incubated with 100 μl horseradish peroxidase-conjugated polyclonal rabbit-anti mouse immunoglobulin antibodies (P0260; Dako, Glostrup, Denmark) diluted 1:1000 in washing buffer. Finally, the plates were washed three times in washing buffer and incubated with 0.4 mg/ml *o*-phenylene-diamine (Kem-En-Tec, Taastrup, Denmark) in developing buffer (35 mM citric acid, 65 mM Na_2_PO_4_, pH 5.0). After 10 min, the colour reaction was stopped with 1.0 M H_2_SO_4_, and the optical density (OD) was measured at 490–650 nm using the SPECTROstar nano Microplate Reader (BMG Labtech, Ortenberg, Germany). Data were processed using MARS software (BMG Labtech).

### Assay for cell-mediated PAD activity

Isolated leucocytes or MNCs were added to microtitre wells coated with fibrinogen, washed and blocked as already described, and incubated for 180 min under agitation at RT. Purified cells were diluted 1:1 with RPMI 1640 to a final concentration of 5 % AB serum and, when relevant, 15 nM phorbol 12-myristate 13-acetate (PMA) and 50 μM 2-AAPA (which were incubated for 20 min with cells prior to stimulation). Cells were removed by washing four times in PBS and 0.05 % Tween-20, and plates were developed as already described.

### PAD2 measurement

PAD2 was measured using an in-house ELISA, as described previously [22]. SF was diluted 1:10 as described in [23] and cell supernatants were diluted 1:1 with PBS containing 0.05 % Tween-20, pH 7.4.

## Results

### Reduction of PADs is a prerequisite for enzyme activity

**Fig. 1:**
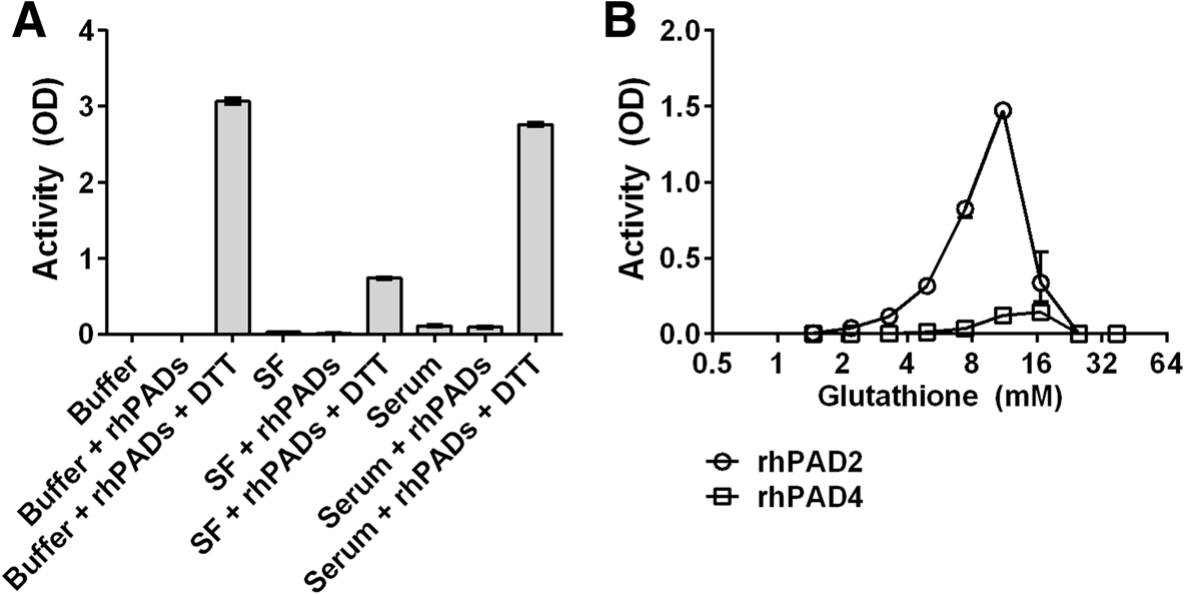
Requirements of PAD for reducing agents. **a** Microtitre plates coated with human fibrinogen were incubated for 3 h at RT with CaCl_2_-containing buffer alone, a pool of synovial fluid (*SF*) from four patients with RA (33 % v/v in buffer) or a pool of serum from five healthy donors (33 % v/v in buffer). rhPAD2 and rhPAD4, mixed at a molar ratio of 1:1, and 1 mM dithiothreitol (*DTT*) were added as indicated. The catalytic activity of rhPADs was measured by an ELISA detecting citrullination of fibrinogen by means of the mAb 20B2, and is expressed as optical density (*OD*). **b** Individual activities of rhPAD2 and rhPAD4 at various concentrations of reduced glutathione (GSH) in buffer containing 10 mM CaCl_2_. All data represent the mean and range of duplicate measurements. *rhPAD* recombinant human peptidylarginine deiminase

PAD activity was determined in microtitre plates containing immobilized human fibrinogen, using an antibody specifically reactive with citrullinated fibrinogen. In the presence of DTT (1 mM in Tris–HCl buffer containing 10 mM CaCl_2_) as a reducing agent, we observed ample citrullination of human fibrinogen by rhPAD2 and/or rhPAD4 (Fig. 1a). No citrullination was observed when a pool of SF from four RA patients was used, although we have previously shown that SF contained PAD2 and 1.5–2.5 mM calcium [12, 23]. The addition of rhPAD2 and rhPAD4 to SF did not result in detectable fibrinogen citrullination, except when DTT was also present. A similar pattern was observed for a pool of sera from five healthy donors.

### GSH-mediated reduction can activate PADs

To investigate whether GSH could substitute for DTT as a reducing agent, we examined the enzymatic activity of rhPAD2 and rhPAD4 over a range of GSH concentrations and at a fixed CaCl_2_ concentration of 10 mM (Fig. 1b). Catalytic activity required GSH concentrations above 1 mM, reached a peak around 10–15 mM and declined with further increasing GSH concentrations. At GSH concentrations above 25 mM, no PAD activity was observed. In contrast, the activity of both rhPAD isoforms reached a plateau at DTT concentrations between 1 mM and 50 mM (data not shown). As shown previously with DTT as a reducing agent [12], PAD2 showed higher efficacy than PAD4 in the employed assay (Fig. 1b).

### Calcium is required for GSH-mediated PAD activity

**Fig. 2:**
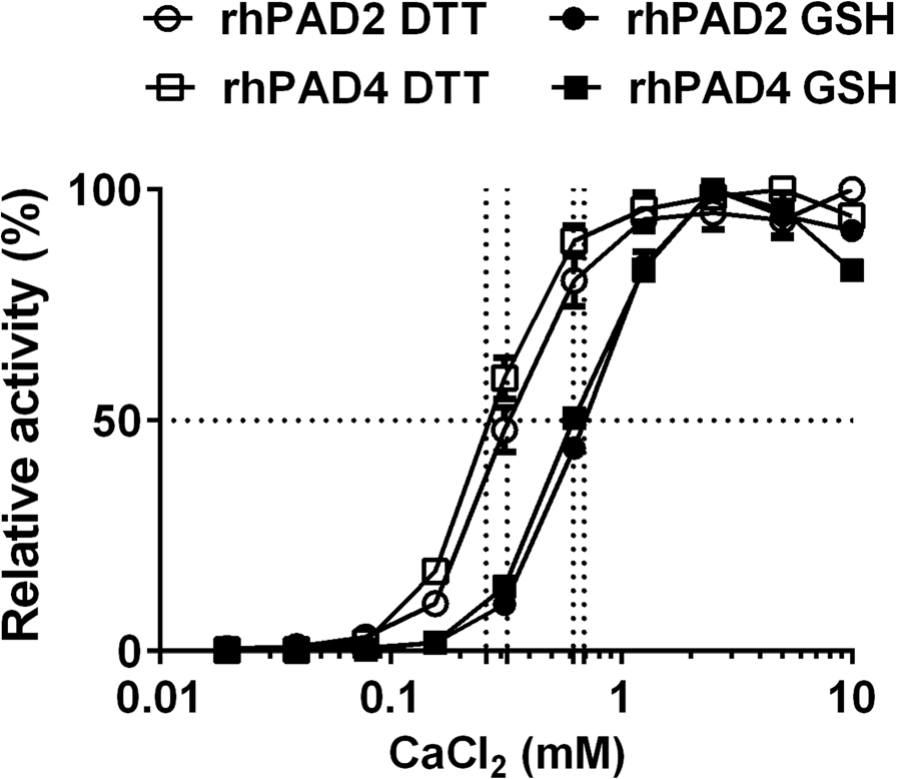
Calcium dependency of PAD reduced by glutathione. Microtitre plates were coated with human fibrinogen and incubated for 3 h at RT with rhPAD2 (300 ng/ml) or rhPAD4 (3000 ng/ml) and either 1 mM dithiothreitol (*DTT*) (*open symbols*) or 10 mM reduced glutathione (*GSH*) (*closed symbols*) in the presence of CaCl_2_ at concentrations ranging from 20 μM to 10 mM. Citrullination of fibrinogen was measured using mAb 20B2 as the detecting antibody. Activity of PAD is shown as the percent of maximal activity for each enzyme, expressed as the mean and range of duplicate measurements. *rhPAD* recombinant human peptidylarginine deiminase

As expected, the catalytic activity of both rhPAD2 and rhPAD4 was calcium dependent, irrespective of whether GSH or DTT was used as a reducing agent (Fig. 2). Higher calcium concentrations were required to obtain half-maximal activities in the presence of GSH than in the presence of DTT; 0.69 mM versus 0.32 mM CaCl_2_, respectively, for PAD2, and 0.62 mM versus 0.26 mM CaCl_2_, respectively, for PAD4. The activity of rhPADs in Tris–HCl buffer containing 10 mM CaCl_2_ was around 8-fold lower in the presence of 10 mM GSH than in the presence of 1 mM DTT (data not shown).

### PAD activity in SF is dependent on reducing conditions

**Fig. 3:**
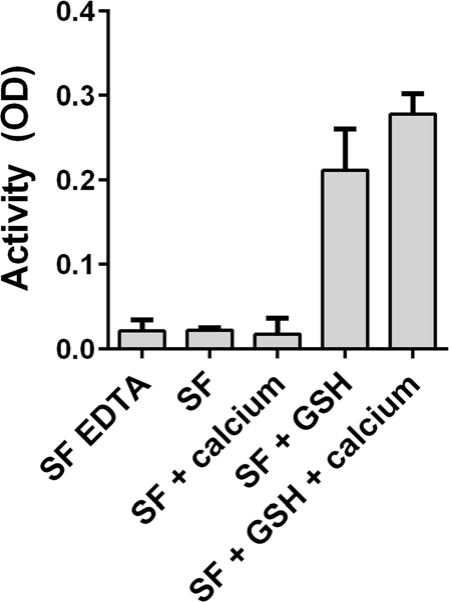
Reduced glutathione (*GSH*) induces PAD activity in synovial fluid (*SF*). Microtitre plates were coated with human fibrinogen and incubated for 3 h at RT with a pool of SF from five RA patients supplemented with different combinations of CaCl_2_ (10 mM), GSH (10 mM) or EDTA (25 mM). Citrullination of fibrinogen was measured using mAb 20B2 as the detecting antibody. Bars and error bars represent the mean and SEM of triplicate measurements

To examine the PAD-reducing capacity of GSH in a physiologically relevant setting, we examined the catalytic activity of native PAD enzymes contained in a pool of SF from five RA patients in the presence and absence of GSH. ELISA verified that the pool contained PAD, the concentration of PAD2 being 18 ng/ml. As shown in Fig. 3, pure SF as well as SF supplemented with 10 mM CaCl_2_ failed to exhibit catalytic activity above that observed in negative controls containing the calcium chelator EDTA, which blocks PAD activity. However, when SF was supplemented with 10 mM GSH in the presence or absence of exogenously added CaCl_2_, substantial citrullination was observed.

### Enzymatic activity of PAD released from stimulated leucocytes

**Fig. 4:**
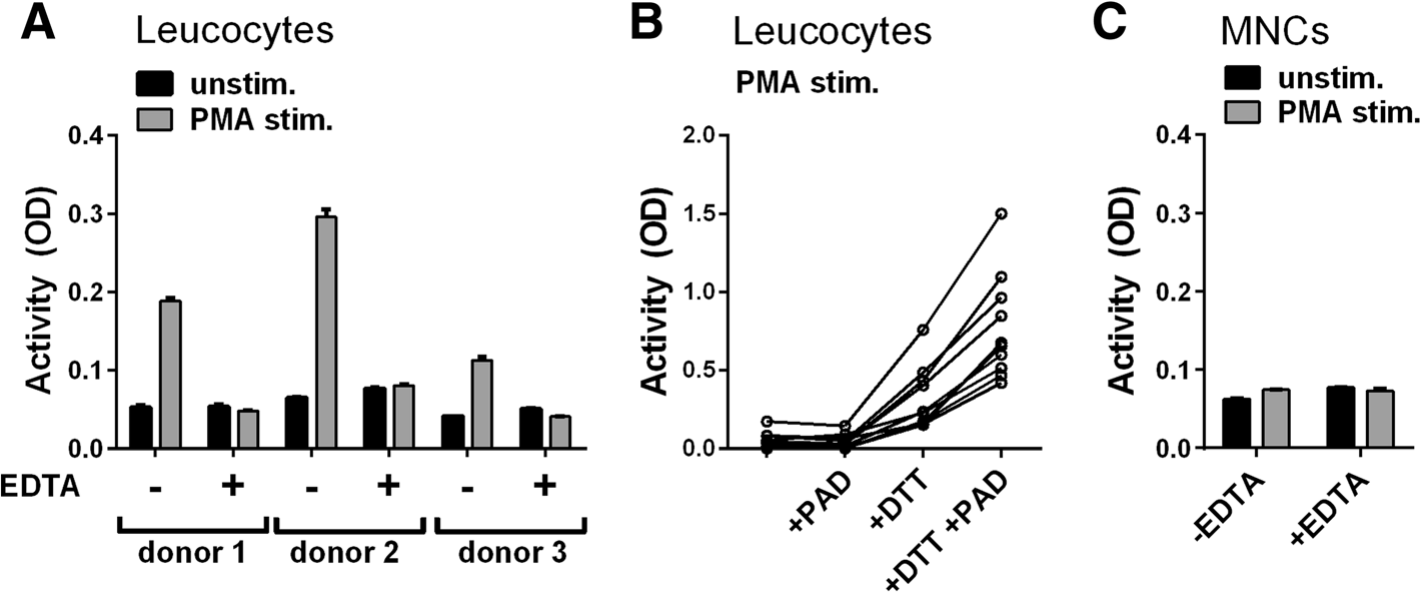
Release of catalytically active peptidylarginine deiminase (*PAD*) from PMA-stimulated granulocytes. **a** Microtitre plates coated with human fibrinogen were incubated for 3 h at RT with blood leucocytes resuspended in RPMI medium containing 5 % normal human serum with or without 25 mM EDTA. Cells were stimulated with PMA (15 nM) and subsequent citrullination of fibrinogen was measured using mAb 20B2 as the detecting antibody. Average and upper range of duplicate optical density (*OD*) measurements shown for three donors. **b** Enzymatic activity of PMA-stimulated leucocytes from 10 individual donors was examined in a similar manner in the presence and absence of exogenous rhPAD2 (300 ng/ml) and/or 1 mM dithiothreitol (*DTT*). Shown are averages of duplicate measurements after subtraction of background values obtained in the presence of EDTA. **c** Using the same procedure, mononuclear cells (*MNCs*) from donor 1 in **a** were assessed. Average and upper range of duplicate OD measurements are shown. *PMA* phorbol 12-myristate 13-acetate

To examine whether PAD released from cells is catalytically active, we cultivated blood leucocyte cells in microwells coated with fibrinogen to allow citrullination in situ (Fig. 4a). No catalytic activity was observed in wells containing unstimulated cells, but stimulation of the leucocytes with PMA resulted in substantial citrullination of fibrinogen (Fig. 4a, grey columns). As expected, this signal could be inhibited by EDTA. Addition of exogenous rhPAD2 did not further increase the catalytic activity in the wells, which was tested in experiments involving leucocytes from 10 different donors (Fig. 4b), indicating that the requirements for PAD activity were not met in the medium. Addition of DTT enhanced activity of the released PAD and rendered the exogenously added rhPAD2 active (Fig. 4b). Taken together, these data suggest that stimulation of leucocytes by PMA leads to the release of PAD, which is temporarily enzymatically active and loses its activity in the oxidative environment.

In contrast with total leucocytes, purified MNCs did not show PAD activity upon stimulation (Fig. 4c), suggesting that granulocytes were the main providers of catalytically active PAD (Fig. 4a). In agreement with the finding that exogenous rhPAD2 did not enhance the catalytic activity in wells containing PMA-stimulated cells, rhPAD2 added to supernatants from the unstimulated or PMA-stimulated cells was not enzymatically active unless supplemented with GSH (data not shown). The highest activity was observed at GSH concentrations around 20–25 mM, and no activity was observed at a concentration of 50 mM (data not shown).

**Fig. 5:**
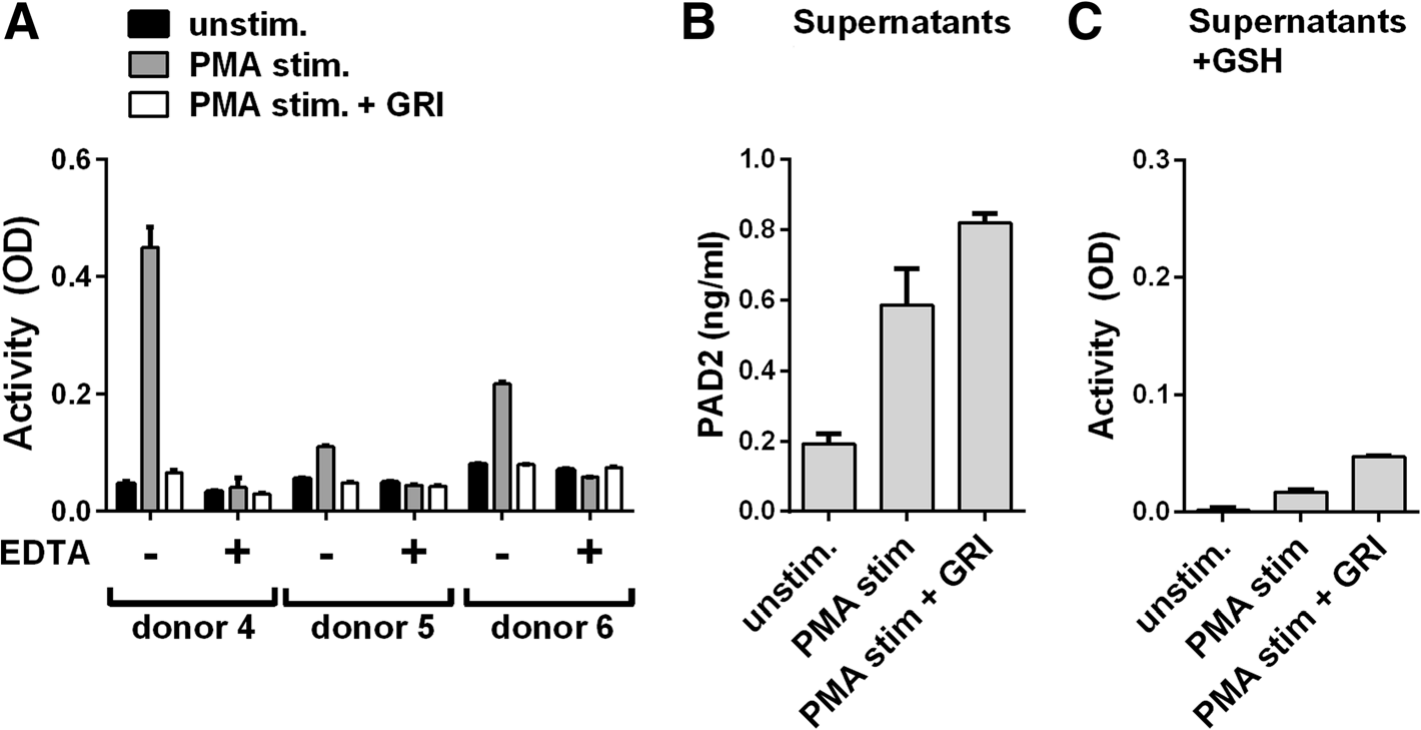
Effect of glutathione reductase inhibition on the catalytic activity of peptidylarginine deiminase (*PAD*) released from cells. **a** Microtitre plates coated with human fibrinogen were incubated for 3 h at RT with leucocytes isolated from three healthy donors in media without PMA, media with 15 nM PMA alone or media with 15 nM PMA in combination with 50 μM of the glutathione reductase inhibitor (*GRI*) 2-AAPA. Citrullination of fibrinogen was detected using mAb 20B2 and optical density (*OD*) values are shown as the average and upper range of triplicate measurements. **b** PAD2 concentration in cell supernatants is shown as the average and upper range of duplicate measurements. **c** Catalytic activity of PAD in cell supernatants supplemented with 5 mM CaCl_2_ and 25 mM of reduced glutathione (*GSH*) shown as the average and upper range of duplicate OD measurements using mAb 20B2 as a detector of citrullinated fibrinogen. *PMA* phorbol 12-myristate 13-acetate

To determine whether PADs released from stimulated leucocytes (Fig. 4a) had gained catalytic activity through reduction by endogenous GSH, we stimulated leucocytes with PMA in the presence of a selective GRI (2-AAPA) [24] (Fig. 5a). Inclusion of the GRI abrogated enzymatic activity signals to those observed in the presence of EDTA. Importantly, GRI did not prevent PAD from being released. Thus, inclusion of GRI diminished neither the PAD2 concentration (Fig. 5b) nor the PAD activity (Fig. 5c) of the supernatants. No activity was observed in supernatants without exogenously added GSH (data not shown).

## Discussion

The physiological agent(s) responsible for reducing PADs to their physiologically active state have not been identified. In general, PAD activity studies in vitro have used DTT or a related compound not present in nature as a reducing agent. Thus, enzymatic activity of PADs contained in SF [12, 23], in cell lysates [25] or released by cells [26, 27] has generally not been examined under physiological conditions, because DTT has been included in the experiments. We hypothesized that GSH is a natural reducing agent required for PADs to be enzymatically active.

Indeed, enzymatic activity of rhPAD2 and rhPAD4 was observed at GSH concentrations corresponding to those found in cytosol (i.e. around 4.5 mM) [15]. To our knowledge, intracellular GSH levels exceeding 15 mM have not been observed in vivo, and therefore the diminished PAD activity observed at high concentrations of exogenously added GSH in this study may not be physiologically relevant.

We did not observe any PAD activity in pooled SF from RA patients without addition of GSH or DTT. Spengler et al. [27] recently reported weak PAD activity in pure, freshly obtained SF from untreated RA patients, which was higher than in SF from OA patients, albeit 100-fold lower than the activity in the presence of DTT-containing citrullination buffer. The differences between their observations and ours may rely on their use of a different assay for protein citrullination and, possibly, usage of freshly isolated SFs. The low or absent PAD activity in SF observed here and by others [27] suggests that an essential factor was missing for PADs to function optimally. Our finding that addition of DTT or GSH to SF strongly enhanced PAD activity indicates that a reducing agent(s) is this essential factor.

Circulating proteins produced outside joints (e.g. fibrinogen produced in the liver) are present in a citrullinated form in SF from RA patients [28, 29], suggesting that extracellular citrullination occurs within the joints, where the calcium concentration is sufficiently high for PADs to be active. Our finding that PMA-stimulated leucocytes cultured in microtitre wells were capable of citrullinating fibrinogen coated in wells suggests that the leucocytes either released PAD in reduced form or co-released substance(s) capable of reducing PAD.

Evidence for GSH being critical for reduction of PAD came from the finding that citrullination was abrogated by addition of the highly specific GRI 2-AAPA [24] to cells before incubation in the wells. Extracellular leucocyte PAD most probably originates from granulocytes, because PMA-stimulated MNCs devoid of granulocytes were not capable of citrullinating fibrinogen. The presence of PAD2 and of PAD activity (after addition of GSH) in supernatants from the cultures exposed to 2-AAPA suggested that 2-AAPA did not inhibit PAD release. However, we cannot rule out that 2-AAPA has effects on PAD activity other than that caused by enhancement of the GSSG:GSH ratio. The enzymatic activity of the supernatants was low compared with the activity observed in the presence of cells, suggesting that citrullination of fibrinogen took place in the close vicinity of the cells, where high local concentrations of reduced PADs could be obtained. It is likely that PADs are rapidly oxidized, and thereby inactivated, upon release from granulocytes.

With respect to citrullination of intracellular proteins, the PAD requirement for GSH is clearly met intracellularly [15]. Indeed, intracellularly located citrullinated proteins have been observed in the synovium of RA patients, indicating that calcium concentrations high enough for PADs to be active can be reached intracellularly under certain circumstances [13]. Accordingly, PAD enzymes are known to modulate gene expression [30–32]. It is noteworthy that GSH can also be recruited to the nucleus (e.g. during the early phase of cell proliferation [33]) and is also involved in gene expression [34, 35].

A limitation of this study is that we cannot distinguish between PAD2 and PAD4 activity. Granulocytes are known to release both isoforms upon PMA stimulation [26, 27]. We have shown previously that ~150-fold more rhPAD4 than rhPAD2 is required to citrullinate the epitope recognized by the detecting antibody in the assay used in this study [12], and thus it can be speculated that PAD2 is primarily responsible for the citrullination observed. We cannot exclude an influence of other physiological reducing agents on PAD activation in vivo. Indeed, thioredoxin has been reported to be 5-fold increased in SF of RA patients compared with OA patients [36]. However, the complete abrogation of cell-mediated citrullination by blockade of glutathione reductase suggests that GSH is predominantly responsible for reduction of PAD under normal physiological conditions.

## Conclusion

This study shows for the first time that the physiological reducing agent GSH, at concentrations similar to those present in cytosol, is capable of reducing PADs to a degree that suffices for citrullination to occur. We found that the thiol status of PADs is as important as binding of calcium for PAD’s catalytic activity.

## Abbreviations

2-AAPA: 2-acetylamino-3-[4-(2-acetylamino-2-carboxyethylsulfanylthiocarbonylamino)phenylthiocarbamoylsulfanyl]propionic acid hydrate
ACPA: anti-citrullinated protein antibody
DTT: dithiothreitol
GRI: glutathione reductase inhibitor
GSH: reduced glutathione
GSSG: oxidized glutathione
MNC: mononuclear cell
OD: optical density
PAD: peptidylarginine deiminase
PMA: phorbol 12-myristate 13-acetate
RA: rheumatoid arthritis
rhPAD: recombinant human peptidylarginine deiminase
RT: room temperature
SF: synovial fluid
